# Use of serum squamous cell carcinoma antigen for follow-up monitoring of cervical cancer patients who were treated by concurrent chemoradiotherapy

**DOI:** 10.1186/1748-717X-5-78

**Published:** 2010-09-15

**Authors:** Sang Min Yoon, Kyung Hwan Shin, Joo-Young Kim, Sang Soo Seo, Sang-Yoon Park, Sung Ho Moon, Kwan Ho Cho

**Affiliations:** 1 Research Institute and Hospital, National Cancer Center, 809 Madu 1-dong, Ilsandong-gu, Goyang-si, Gyeonggi-do, 410-769, Republic of Korea; 2 Department of Radiation Oncology, Asan Medical Center, University of Ulsan College of Medicine, Seoul, Republic of Korea

## Abstract

**Background:**

To investigate the significance of monitoring the levels of the serum squamous cell carcinoma antigen (SCC-Ag) for the detection of recurrent disease in patients with cervical cancer treated by concurrent chemoradiotherapy.

**Methods:**

The records of 112 patients with cervical cancer were reviewed. Serum SCC-Ag levels were measured at regular follow-up visits. A SCC-Ag level of 2 ng/mL was considered the upper limit of normal. Biochemical failure was defined as two consecutively increasing SCC-Ag values above normal. Recurrent disease was confirmed by histologic and radiographic studies.

**Results:**

Eighteen patients (16%) developed recurrent disease. Sixteen patients had initially elevated SCC-Ag, post-treatment normalization of SCC-Ag, and tumor recurrence. The SCC-Ag difference (ΔSCC-Ag), defined as the difference between the last value after two consecutively increases above normal and the value immediately before the elevation, had good clinical performance in predicting cancer recurrence. The cutoff value of ΔSCC-Ag was 0.95 ng/mL.

**Conclusions:**

SCC-Ag is a relatively good method for the detection of disease recurrence in patients with cervical cancer who were treated by concurrent chemoradiotherapy.

## Background

Radiotherapy has maintained its place as the cornerstone of therapy for many decades for uterine cervical cancer. Recently, the results of several randomized trials have recommended the concomitant administration of chemotherapy and radiotherapy as a standard treatment for patients with locally advanced cervical cancer [1-3]. Although this combination treatment plays a role in improving disease control, many patients suffer from tumor recurrence during the follow-up period. Therefore, the identification of prognostic factors associated with disease course and outcome following chemoradiotherapy may help to guide the development of more effective treatments and prevent tumor recurrence.

Over the past decade, several serum markers have been investigated to search for additional prognostic parameters that could be used, to monitor the treatment response, and detect the recurrence in patients with cervical cancer. In particular, the squamous cell carcinoma antigen (SCC-Ag) is the most commonly used tumor marker for cervical cancer. SCC-Ag is a sub-fraction of the tumor antigen TA-4, a 48 kDa glycoprotein first isolated by Kato and Torigoe [4]. This antigen is present in normal cervical epithelium, but has higher expression in cervical neoplasms [5]. With the development of a sensitive radioimmunoassay, this marker can be readily detected in the serum and is now considered a valuable tool for monitoring cervical cancer. Serum SCC-Ag levels correlate with the extent of disease [6-8], response to radiotherapy [9], response to chemotherapy [10,11], and can be used to predict survival and tumor recurrence during follow-up [12-16]. Several studies have reported the use of serial SCC-Ag data for post-therapeutic monitoring. In these reports, 70-86% of cervical cancer patients with recurrent disease had elevated SCC-Ag levels at some time during follow-up [7,8,11,14,16]. However, few studies have focused on cervical cancer patients treated by concurrent chemoradiotherapy (CCRT), which is now considered the standard treatment for locally advanced cervical cancer.

In the present study, we investigated the potential use of SCC-Ag as a marker for predicting tumor recurrence in uterine cervical cancer patients treated with CCRT.

## Methods

Between July 2001 and February 2004, 124 previously untreated women with the International Federation of Gynecology and Obstetrics (FIGO) stage IB-IV uterine cervical cancer were entered in a CCRT protocol at the National Cancer Center (Goyang, Gyeonggi, Republic of Korea). Twelve cases that lacked regular SCC-Ag determinations in the follow-up period were excluded from the study. The pre-treatment evaluation consisted of a complete medical history, physical examination, full blood counts, biochemical profile, serum SCC-Ag, chest radiography, intravenous pyelogram, cystoscopy, rectosigmoidoscopy, magnetic resonance imaging (MRI) or computed tomography (CT) scan, and/or [18F]-flouro-2-deoxy-D-glucose positron emission tomography (FDG-PET). Written informed consent was obtained from all the patients.

All patients were given external beam radiotherapy (EBRT) with 15 MV X-rays from a linear accelerator (Varian Clinac 2100CD; Varian, Palo Alto, CA, USA). EBRT was administered to the whole pelvic region using a four-field box technique or parallel opposed anterior-posterior beams. The radiation field included the primary tumor, uterus, paracervical, parametrial and uterosacral regions, and the pelvic lymph nodes. The para-aortic lymph nodes were also included if metastasis was diagnosed during pre-treatment imaging study. The radiation dose administered to the whole pelvis was 41.4-45 Gy (median 45 Gy), given in daily doses of 1.8 Gy, five fractions per week. An additional boost of 5.4-21.4 Gy (median 10 Gy) was given to the gross residual tumor and involved the parametrium without any midline shielding. The dose to the para-aortic area was 45 Gy, with or without an additional booster dose of 10 Gy. Intracavitary brachytherapy was administered twice weekly. Fletcher-Suit afterloading applicators were used for high-dose-rate brachytherapy with an Iridium-192 source (Microselectron^®^; Nucletron, Veenendaal, The Netherlands). The brachytherapy dose for each insertion was 4 or 5 Gy at point A, and the total dose of brachytherapy was 24-35 Gy (median 24 Gy). All patients also were given concurrent chemotherapy, which consisted of weekly doses of 40 mg/m^2 ^i.v. cisplatin for four to six cycles.

After completion of CCRT, all patients were given clinical examinations and assayed for serum SCC-Ag during the follow-up visits. A radiation oncologist and a gynecologist followed the patients for one month after treatment, then every three months for the first two years, and every 3-6 months thereafter. The follow-up intervals varied for patients suspected of having recurrent disease, based on individual situations. Patients with suspicious symptoms, such as signs at the physical examination or an elevated serum SCC-Ag level during follow-up periods, were given additional tests (histological examination, abdomino-pelvic CT, pelvic MRI, FDG-PET, etc.) to confirm the presence of recurrent diseases.

Serum SCC-Ag levels were measured using an immunoradiometric assay with a commercially available kit (SCC-RIABEAD; SRL Inc., Tokyo, Japan). A measurement of 2 ng/mL was considered the upper limit of normal. Biochemical failure was defined as two consecutively increasing SCC-Ag values above the normal limits. For further statistical analysis, after two consecutive elevated SCC-Ag readings, the delta (Δ) SCC-Ag was defined as the difference between the final value and the value immediately before the elevation.

Logistic regression analysis was used to estimate the probability of tumor recurrence from ΔSCC-Ag. P values less than 0.05 were considered significant. A receiver operating characteristic (ROC) curve was used to define the optimal cutoff point for the SCC-Ag values relative to the probability of a recurrence.

## Results

**Figure 1:**
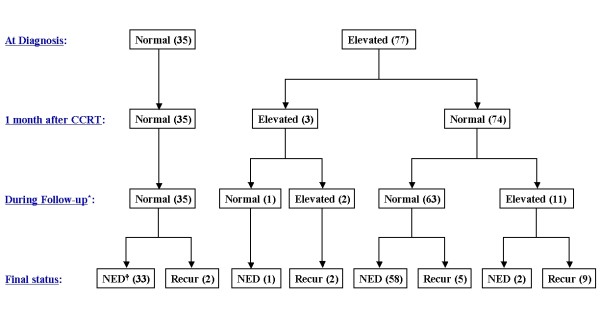
**A diagram of the follow-up results and the change in the SCC-Ag values in all patients**.* Elevated serum SCC-Ag level means a biochemical failure that is defined as two consecutively increasing tumor marker values above the normal limits, † No evidence of disease.

Table 1 shows the demographic and clinical characteristics of the 112 enrolled patients. At diagnosis, 77 patients had elevated serum SCC-Ag, corresponding to an overall pre-treatment sensitivity of 68.8% (77/112). Most patients (96.1%) had normalized SCC-Ag levels at one month after completion of CCRT. The median follow-up duration was 39 months (range, 16 - 55 months). Figure 1 shows the follow-up results.

**Table 1:** Patient characteristics

Variables	
Age -- years	
Median	55
Range	22-78
FIGO stage -- No. (%)	
IB	11 ( 9.8)
IIA	13 (11.6)
IIB	66 (58.9)
III/IV	22 (19.7)
Histology -- No. (%)	
Squamous cell carcinoma	102 (91.1)
Adenocarcinoma	7 ( 6.2)
Adenosquamous cell carcinoma	3 ( 2.7)
Pelvic lymph node* -- No. (%)	
Positive	61 (54.5)
Negative	51 (45.5)
Tumor diameter -- No. (%)	
Range (cm)	1-10
≤4 cm	65 (58.0)
>4 cm	47 (42.0)

Abbreviations: FIGO = International Federation of Gynecology and Obstetrics.

* Pelvic lymph node status was diagnosed by imaging study.

After completion of CCRT, 18 patients (16%) experienced tumor recurrence during the follow-up period. Among these 18 patients, 11 had post-treatment SCC-Ag elevations (biochemical failure) before the appearance of clinically evident disease with a median lead-time of 2 months (range, 1 - 15 months). An analysis of all patients (n = 112) indicated that the sensitivity and specificity of elevated post-treatment SCC-Ag levels in association with recurrent disease were 61.1% and 97.9%, respectively. For patients with elevated pre-treatment SCC-Ag only (n = 77), these values were similar (Table 2).

**Table 2:** Sensitivity, specificity, positive predictive value, and negative predictive value of the tumor maker for predicting a recurrence in all patients* and in patients with elevated pretreatment tumor marker^†^

	SCC-Ag (n = 112)*	SCC-Ag (n = 77)^†^
Sensitivity	11/18 (61.1%)	11/16 (68.8%)
Specificity	92/94 (97.9%)	59/61 (96.7%)
PPV	11/13 (84.6%)	11/13 (84.6%)
NPV	92/99 (92.9%)	59/64 (92.2%)

Abbreviations: SCC-Ag = squamous cell carcinoma antigen; PPV = positive predictive value; NPV = negative predictive value.

**Figure 2:**
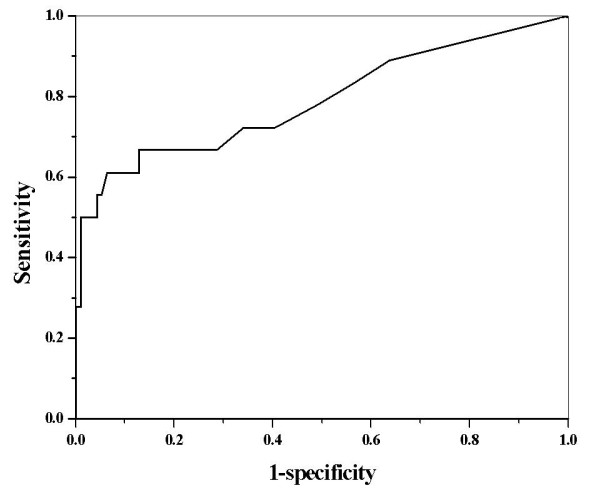
**Receiver operating characteristic curve for ΔSCC-Ag in predicting the probability of a recurrence in all patients**.

**Figure 3:**
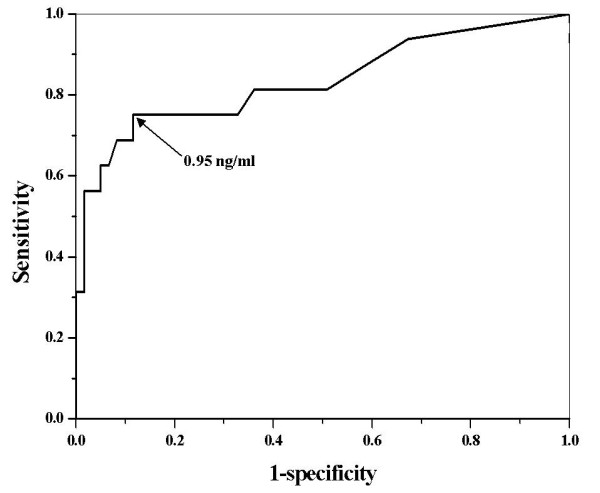
**Receiver operating characteristic curve for ΔSCC-Ag in predicting the probability of a recurrence in patients with elevated pre-treatment tumor markers**.

Next, we performed logistic regression of all patients (n = 112) to estimate the probability of tumor recurrence based on ΔSCC-Ag measurements. Our results indicate that ΔSCC-Ag was a significant predictor of tumor recurrence (p = 0.001). Then, we performed ROC analysis of ΔSCC-Ag to determine the optimal levels for predicting the probability of tumor recurrence (Figure 2). The area under the ROC curve of ΔSCC-Ag, which is considered to indicate the accuracy of the test, was 0.78. The predictability of recurrence was analyzed for patients who had an increase in the level of pre-treatment SCC-Ag (n = 77). Logistic regression analysis also indicated that ΔSCC-Ag was a significant predictor of tumor recurrence (p = 0.002). Figure 3 shows the ROC curve for ΔSCC-Ag in patients with elevated pre-treatment SCC-Ag (n = 77). These results indicate the area under the ROC curve of ΔSCC-Ag was 0.83 and that a ΔSCC-Ag value of 0.95 ng/mL was the optimal cutoff level for prediction of tumor recurrence. These mean that the true positive and false positive rates of tumor recurrence were 75% and 11% (respectively) when the difference between the last value after two consecutive increases above normal and the value immediately before the elevation was 0.95 ng/mL.

## Discussion

Our analysis of cervical cancer patients treated with CCRT indicated that the sensitivity and specificity of two consecutive increases in serum SCC-Ag for predicting tumor recurrence were 61.1% and 97.9%, respectively. These results are comparable to previously reported results. For example, several studies of cervical cancer patients showed that an elevated serum SCC-Ag level was associated with 70-92% rate of recurrent tumors [8,11,14-19]; in addition, the specificity of SCC-Ag during the follow-up period was quite high, varying from 95 to 98% [7,16,20]. According to our ROC analysis, the area under the ROC curve indicated the ΔSCC-Ag was 0.83 for patients who had elevated pre-treatment SCC-Ag. Therefore, our results indicate that ΔSCC-Ag had good clinical performance in detection of recurrent disease.

As described above, we defined biochemical failure as two consecutive SCC-Ag values above normal. There are no standard criteria used to define biochemical failure in cervical cancer, and many previous studies have simply defined failure as an increase of tumor markers above normal. However, Duk et al. [20] reported that the predictive value of a single elevation of serum SCC-Ag level for early detection of recurrence was only 48.6%, but that the predictive value was 76% when two consecutive elevated serum SCC-Ag levels were used. Furthermore, they reported that serum SCC-Ag in the second sample was considerably higher in patients who had tumor relapse than in those with no evidence of relapse. Similarly, 10 of our patients had only once instance of elevated serum SCC-Ag, with normalization at subsequent follow-ups. None of these 10 patients had any evidence of disease at the final follow-up. Therefore, we suggest use of additional measurement of SCC-Ag in patients who have a single SCC-Ag elevation above the normal limits. The use of sequential serum determinations appears to increase the sensitivity and specificity of serum SCC-Ag in predicting tumor recurrence.

The results reported here and those of others indicate that routine surveillance of serum SCC-Ag during follow-up can be used to predict tumor recurrence, but it is unclear if the early detection of recurrence will lead to better survival. The major aim of follow-up surveillance in cervical cancer is the early recognition of a recurrence, based on the presumption that early detection of tumor recurrence may allow effective salvage therapy [18]. However, the clinical relevance of detecting early recurrence of cervical cancer is controversial. Esajas et al. [18] showed that routine assessment of serum SCC-Ag in follow-up resulted in the earlier detection of a recurrence in a small proportion of patients, but did not appear to contribute to better survival. Chan et al. [17] reported similar results, in that the addition of SCC-Ag monitoring provided no advantage over a regular follow-up with a clinical examination. They concluded that posttreatment SCC-Ag monitoring was not cost-effective because there was no curative treatment for distant spread of disease. Other studies, however, have suggested that follow-up SCC-Ag measurements may improve survival. Chou et al. [21] studied the clinical features of isolated para-aortic lymph node recurrence after definitive radiotherapy and reported a good correlation between lower SCC-Ag levels (SCC-Ag ≤4 ng/mL) at recurrence and disease-free survival. They concluded that periodic surveillance with this tumor marker and imaging studies allowed the early detection and implementation of salvage therapy. Forni et al. [22] reported that a simplified diagnostic approach that included the SCC-Ag assay and a gynecologic examination allowed early detection of cervical cancer recurrence with a very favorable cost-effective profile. Moreover, other recent studies reported that the use of FDG-PET in patients with asymptomatic SCC-Ag elevation allowed an earlier diagnosis of recurrence, with promising effects on survival [23,24]. Therefore, further study will be needed to confirm the value of routine surveillance of SCC-Ag in improving survival rate from recurrent cervical cancer after earlier detection.

## Conclusions

In summary, measurement of ΔSCC-Ag provided good clinical performance in the detection of recurrent uterine cervical cancer following CCRT. We suggest that clinicians consider performing routine measurement of SCC-Ag during the follow-up of such patient. However, our results should be interpreted with some caution, because our overall recurrence rate was very low and the follow-up period was relatively short. Therefore, a more comprehensive, large-scale study should be performed to confirm our results.

## List of abbreviations

SCC-Ag: squamous cell carcinoma antigen; CCRT: concurrent chemoradiotherapy; FIGO: International Federation of Gynecology and Obstetrics; MRI: magnetic resonance imaging; CT: computed tomography; FDG-PET: [18F]-flouro-2-deoxy-D-glucose positron emission tomography; EBRT: external beam radiotherapy; ROC: receiver operating characteristic.

## Competing interests

The authors declare that they have no competing interests.

## Authors' contributions

Each author had participated sufficiently in the work to take public responsibility for appropriate portions of the study. SMY participated in research design, coded the patient database, conducted the analysis and wrote the manuscript draft. KHS designed the project, analyzed the data and revised the manuscript. KHC contributed to study conception and design. SHM and JYK helped with the database and data analysis. SSS and SYP provided additional guidance and support for this research. All authors read and approved the final version of the manuscript.
